# Effects of extracellular adhesion molecules on immune cell mediated solid tumor cell killing

**DOI:** 10.3389/fimmu.2022.1004171

**Published:** 2022-10-27

**Authors:** Seong-Eun Kim, Suji Yun, Junsang Doh

**Affiliations:** ^1^ Department of Mechanical Engineering, Pohang University of Science and Technology (POSTECH), Pohang, Gyeongbuk, South Korea; ^2^ Interdisciplinary Program for Bioengineering, Seoul National University, Seoul, South Korea; ^3^ Department of Materials Science and Engineering, Research Institute of Advanced Materials, Institute of Engineering Research, Bio-MAX Institute, Soft Foundry Institute, Seoul National University, Seoul, South Korea

**Keywords:** cytotoxicity assay, single cell array, microwell assay, live cell imaging, extracellular adhesion molecules, NK cell, T cell, cancer immunotherapy

## Abstract

Adoptive cell therapy (ACT) using *ex vivo* engineered/expanded immune cells demonstrated poor efficacy against solid tumors, despite its great success in treating various hematopoietic malignancies. To improve ACT for solid tumors, it is crucial to comprehend how the numerous components of the tumor microenvironment (TME) surrounding solid tumor cells influence killing ability of immune cells. In this study, we sought to determine the effects of extracellular adhesion provided by extracellular matrix (ECM) of TME on immune cell cytotoxicity by devising microwell arrays coated with proteins either preventing or promoting cell adhesion. Solid tumor cells in bovine serum albumin (BSA)-coated microwells did not attach to the surfaces and exhibited a round morphology, but solid tumor cells in fibronectin (FN)-coated microwells adhered firmed to the substrates with a flat shape. The seeding densities of solid tumor cells and immune cells were tuned to maximize one-to-one pairing within a single microwell, and live cell imaging was performed to examine dynamic cell-cell interactions and immune cell cytotoxicity at a single cell level. Both natural killer (NK) cells and T cells showed higher cytotoxicity against round tumor cells in BSA-coated microwells compared to flat tumor cells in FN-coated microwells, suggesting that extracellular adhesion-mediated firm adhesion of tumor cells made them more resistant to immune cell-mediated killing. Additionally, NK cells and T cells in FN-coated microwells exhibited divergent dynamic behaviors, indicating that two distinct subsets of cytotoxic lymphocytes respond differentially to extracellular adhesion cues during target cell recognition.

## Introduction

Adoptive cell therapy (ACT) using *ex vivo* cultured/engineered immune cells is an emerging therapy for cancer treatment since the recent success of chimeric antigen receptor (CAR) T cell therapy (1, 2). While CAR-T cell therapy has been tremendously successful in clinical trials and is now being used to treat refractory cancer patients, it still faces a number of challenges (3). For example, T cells from cancer patients must be used to prepare CAR-T cells because T cells from donors can cause severe allogeneic immune responses, necessitating weeks of manufacturing time and high manufacturing expenses (4). While genome editing tools are applied to T cells to minimize allogeneic immune responses (5), other subsets of lymphocytes such as natural killer (NK) cells that efficiently kill tumor cells but exhibit minimal adverse effects in allogeneic settings are considered to develop allogeneic off-the-shelf immune cell therapeutics (6). In addition, CAR-T cell therapy has been successful for B cell-originated blood cancers such as leukemia, lymphoma, and multiple myeloma, but its application for solid tumors has not been straightforward (7).

One of the most critical assays to develop immune cells for ACT-based cancer treatment is the cytotoxicity assay, which measures the ability of immune cells to kill tumor cells. Standard cytotoxic assays are performed by mixing immune cells and tumor cells in test tubes or round-bottomed well plates, and measuring tumor cell death within them. By quantifying the release of radioactive materials such as ^51^Chromium or fluorescence dyes such as Calcein preloaded in tumor cells, or the incorporation of fluorescence dyes such as propidium iodide (PI) into the dead tumor cells, immune cell-mediated tumor cell death is measured (8, 9). While these assays allow high throughput assessment of immune cells’ ability to kill tumor cells, the results may not be fully translated to *in vivo* therapeutic efficacy because the microenvironment surrounding tumor cells also influences immune cell-mediated tumor cell death, particularly in solid tumors (10, 11).

To overcome this limitation, 3D microfluidic chips mimicking complex tumor microenvironment (TME) such as hypoxia, vascularization, extracellular matrix densification, and immune suppressor cells have been developed and used to evaluate the performance of *ex vivo* engineered immune cells (12–16). While these 3D models enable cytotoxicity assay to be performed in a 3D TME context, complex heterogeneous 3D environments preclude detailed mechanistic analysis at the single cell level. In contrast, microwell arrays that confine defined number of tumor cells and immune cells within a microwell allow live cell imaging-based single cell level detailed analysis of tumor cell-immune cell interactions (17–21), whereas lack complex 3D TME.

Herein, we developed microwell arrays coated with either bovine serum albumin (BSA), which prevent cell adhesion, or fibronectin (FN), which promotes cell adhesion, thus allow us to control extracellular adhesion environments of tumor cells. Tumor cell adhesion on extracellular matrix (ECM) plays a central role in various stages of cancer metastasis (22–24): tumor cells in mesenchymal phenotype, formed by epithelial-mesenchymal transition (EMT), typically exhibit strong adhesion on ECMs and slowly migrate on ECMs, whereas tumor cells in amoeboid phenotype, formed by mesenchymal-amoeboid transition (MAT), relatively weakly adhere on ECMs and quickly migrate through ECM pores. Tumor cells undergone intravasation become circulating tumor cells (CTCs) that do not rely on ECM adhesion, but the CTCs needs to regain ECM adhesion after extravasation to settle in metastatic tissues (25–27). Using these microwell arrays, we investigated the effects of extracellular adhesion on immune cell-mediated solid tumor cell killing. By directly observing the interaction between immune cells, such as NK cells and T cells, and solid tumor cells using live cell imaging, we demonstrated that extracellular adhesion modulated not only the susceptibility of solid tumor cells to immune cell-mediated killing, but also the immune cell dynamics during their target cell recognition.

## Results

### Bulk population level assessment of immune cell cytotoxicity against surface attached vs. suspended solid tumor cells

**Figure 1:**
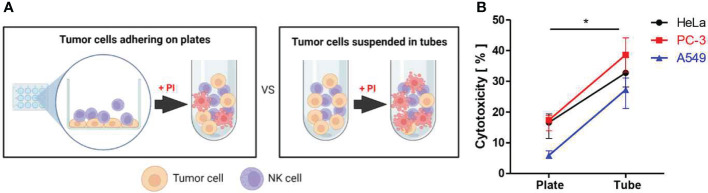
Assessment of NK cell cytotoxicity against solid tumor cells adhering on plates vs. suspended in tubes. **(A)** Schematic illustration of experimental settings. **(B)** Effects of substrate adhesion of various solid tumor cells on NK cell mediated cytotoxicity. Mann-Whitney test was used, * < 0.05. Created with BioRender.com.

Cytotoxicity assays are typically conducted by mixing immune cells and tumor cells in suspension, regardless of tumor cells are originated from solid tumors or not. We first asked whether adhesion status of solid tumor cells influenced immune cell cytotoxicity by comparing immune cell cytotoxicity against suspended and surface attached solid tumor cells as schematically shown in **Figure 1A**. Three different types of solid tumor cell lines including HeLa (cervical cancer), PC-3 (prostate cancer), and A549 (lung cancer) were labeled with 5,6-carboxyfluorescein diacetate succinimidyl ester (CFSE) and placed in either flat-bottom cell culture plates or round-bottom tubes. Cells in flat-bottom plates were incubated for 3 h to allow cells to adhere on the surfaces. NK-92 cells were added to flat-bottom plates, where tumor cells are firmly attached and spread, or round-bottom tubes, where tumor cells are suspended, and incubated for 4 h. Then, propidium iodide (PI) was added to the cell mixture to label dead cells, and flow cytometry analysis was performed. Cytotoxicity was calculated by the percentage of PI^+^ (dead) cells among CFSE^+^ (tumor) cells (**Figure 1B**). NK cells exhibited significantly higher cytotoxicity against tumor cells in suspension than tumor cells attached on the substrates, regardless of tumor cell types, indicating adhesion status of tumor cells influenced NK cell cytotoxicity. However, it is also possible that NK cells mixed with tumor cell suspension exhibited the higher cytotoxicity simple because they had the greater chance of encountering tumor cells than NK cells added to surface-attached tumor cells, due to the higher local cell density.

### Fabrication of microwells that can control tumor cell adhesion status

**Figure 2:**
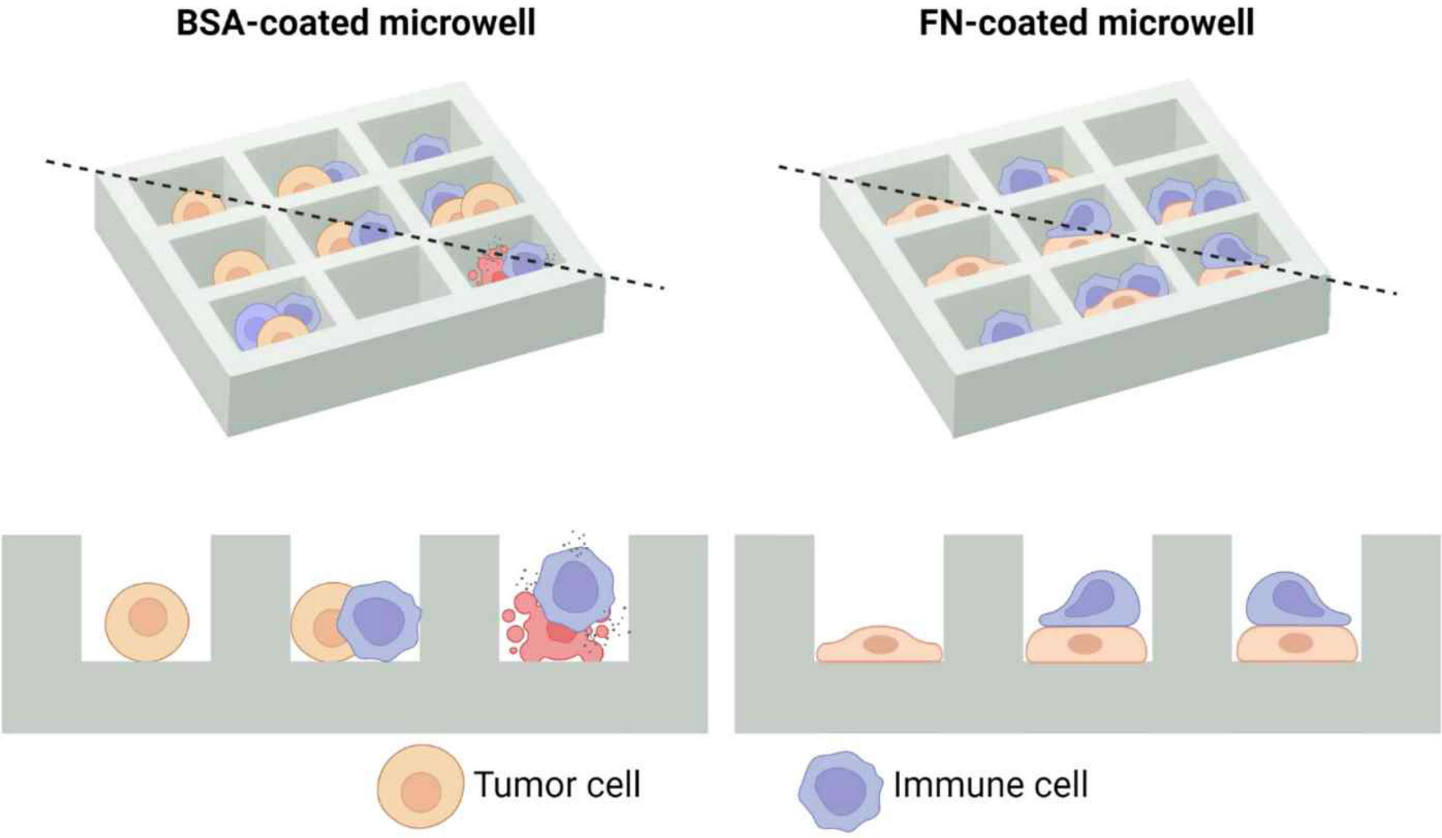
Schematic illustration of microwell arrays that can control adhesion status of solid tumor cells. As bovine serum albumin (BSA) does not allow cell adhesion, solid tumor cells in BSA-coated microwells will have a round morphology, whereas fibronectin (FN) promotes cell adhesion, solid tumor cells in FN-coated microwells will have a flat morphology. Top views are shown in the upper panel, and cross sectional views across the black dashed lines are shown in the lower panel. Created with BioRender.com.

In order to more precisely evaluate how immune cell cytotoxicity against solid tumor cells is influenced by adhesion status of tumor cells, we devised microwell arrays coated with either cell-adhesion or cell-repellent proteins, thus can control adhesion status of tumor cells (**Figure 2**). Using this setting, microwells loaded with one tumor cell and one immune cell can be selected and directly observed by live cell imaging to assess single cell level immune cell cytotoxicity. To implement this idea, microwells were either coated with fibronectin (FN), an adhesion molecules ubiquitously present in extracellular matrix of many different tissues/organs (28, 29), or bovine serum albumin (BSA), a protein widely used to prevent cell-surface interactions (30).

**Figure 3:**
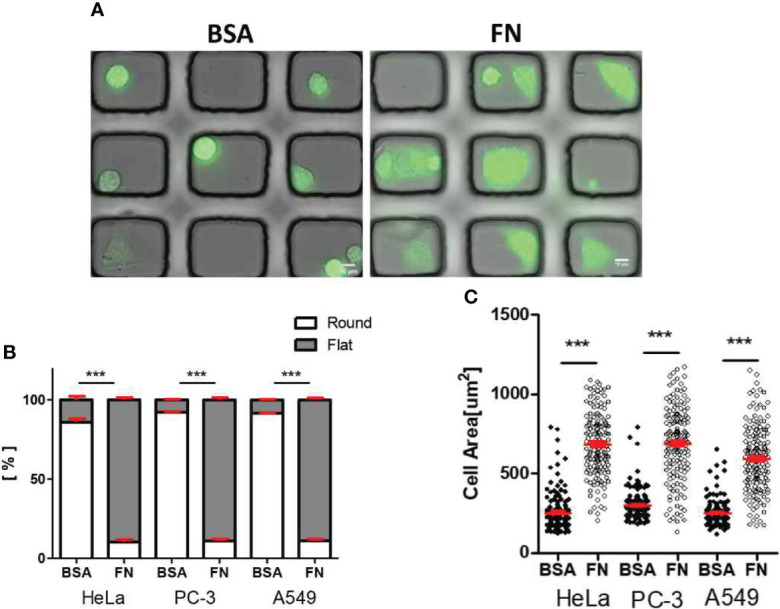
Morphology of solid tumor cells in BSA-coated and FN-coated microwells. **(A)** DIC/fluorescence overlay images of HeLa cells (green) loaded in microwell arrays (microwell Size: 50 μm x 45 μm) either coated with BSA (left) or FN (right). Scale bars: 10 μm. **(B, C)** Effects of microwell coating on morphology **(B)** and cell area **(C)** of various solid tumor cells. Mann-Whitney test was used, *** < 0.001.

Tumor cells labeled with fluorophores were seeded in the microwells, and incubated for 3 h to allow them to adhere on the surfaces. Then, differential interference contrast (DIC) and fluorescence images were acquired to assess cell adhesion and spreading in microwells (**Figure 3A**). In the BSA-coated microwells, 80 ~ 90% of tumor cells failed to adhere on the surfaces and exhibited round shapes, whereas in the FN–coated microwells, ~ 90% of tumor cells adhered on the surfaces and exhibited flat shapes (**Figure 3B**). Consequently, average areas of cells in BSA-coated microwells were two- to three-fold lower than those of cells in FN-coated microwells (**Figure 3C**). These results indicate that we could control adhesion status of tumor cells by microwell surface coating.

### Optimization of one-to-one cell pairing of immune-tumor cells in microwell

**Figure 4:**
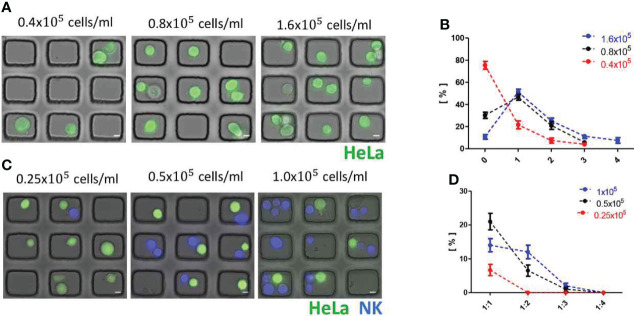
Optimization of one-to-one cell pairing in microwells. **(A)** DIC/fluorescence overlay images of HeLa cell (green)-loaded microwell arrays with various seeding density of HeLa cells. Scale bars: 10 μm. **(B)** Distributions of cancer cell number/microwell. **(C)** DIC/fluorescence overlay images of HeLa cell (green)-NK-92 cell (Blue) loaded microwell arrays with fixed cell seeding density of HeLa cells (0.8 × 10^5^/ml) and various cell seeding density of NK-92 cells. Scale bars: 10 μm. **(D)** Distribution of tumor:NK ratio/microwell. All data representative of three independent experiments.

Next, we optimized the probability to have one-to-one pairing of immune and tumor cells in microwells by varying cell seeding density. Fluorophore-labeled HeLa suspensions with three difference cell densities (0.4 × 10^5^ cells/ml, 0.8 × 10^5^ cells/ml, and 1.6 × 10^5^ cells/ml) were applied on microwell arrays, and DIC and fluorescence images of microwell arrays loaded with the tumor cells were acquired (**Figure 4A**) and analyze cell loading in each microwell (**Figure 4B**). At the lowest cell density (0.4 x 10^5^/ml), ~ 70% of microwells were empty and only ~20% of microwells were occupied by single tumor cells, whereas ~ 50% of single cell occupancy was achieved for the other two cell densities (0.8 × 10^5^/ml and 1.6 × 10^5^/ml). Next, tumor cell density was fixed to 0.8 × 10^5^/ml and NK cell suspensions in three different cell densities (0.25 × 10^5^ cells/ml, 0.5 × 10^5^ cells/ml, and 1.0 x 10^5^ cells/ml) were added to the tumor cell-loaded microwell arrays (**Figure 4C**). For three cell densities tested, the best one-to-one pairing probability (0.21 ± 0.02) was achieved for NK cell density of 0.5× 10^5^ cells/ml (**Figure 4D**). In this way, we optimized seeding densities of tumor cells and immune cells to yield the maximum one-to-one pairing of tumor-immune cells, which is important for the throughputs of the live cell imaging-based cytotoxicity assays.

### Effects of extracellular adhesion on immune cell-mediated cytotoxicity

With this experimental setting, we first assessed the effects of extracellular adhesion of tumor cells on NK cell-mediated cytotoxicity by acquiring time-lapse images of tumor cell-NK cell interactions and analyzing them. Time-lapse microscopy was initiated 15 min after NK cell seeding, and conducted for 5.5 h with a 5 min interval. A motorized stage was used to acquire images in 15 positions in each time interval. Typically, 2 ~ 3 microwells with one–to-one pair were observed in each field of view, thus ~ 40 NK-cancer interactions were analyzed in each experiment.

**Figure 5:**
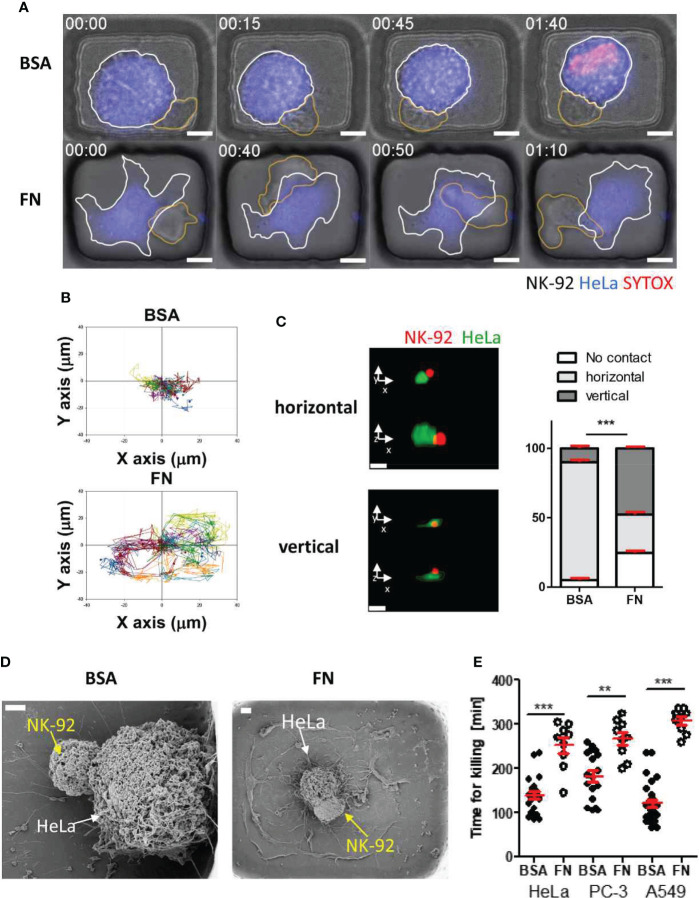
Dynamic behaviors and cytotoxicity of NK cells in microwells coated with BSA or FN **(A)** Representative time-lapse images of NK-HeLa interactions in microwells coated with BSA or FN. Scale bars: 10 μm. **(B)** Representative trajectories of NK cell centroid. For each case, 15 NK cells were traced over 5 h and plotted with the initial NK-tumor contact points as the origin. **(C)** Representative confocal images of NK-92 (red) HeLa (green) pairs in microwells and their distributions in BSA- and FN-coated microwells. Scale bars: 15 μm. **(D)** Representative SEM images of NK-92-HeLa pairs in BSA- and FN-coated microwells. Scale bar: 2 μm. **(E)** Effects of microwell surface coating on time for killing. Mann-Whitney test was used, ** < 0.01, *** < 0.001.

Interestingly, NK cells interacting with tumor cells exhibited completely different behaviors depending on microwell coating proteins [**Figure 5A** and **Movies S1**, **S2** in supplementary information (SI)]: NK cells in BSA-coated microwells formed stable cell-cell contacts with round tumor cells (**Figure 5A** top panel and **Movie S1**), whereas NK cells in FN-coated microwells continuously migrated on flat tumor cells (**Figure 5A** bottom panel and **Movies S2**). These different modes of interactions were evident by the trajectories of individual NK cells (**Figure 5B**): NK cells in BSA-coated microwells made minimal translocation from the origin where initial engagement with a tumor cell occurred, whereas NK cells in FN-coated microwells explored large portions of microwells. Next, cells in the microwells were fixed 2 h after NK cell seeding, and their 3D interactions were observed by confocal (**Figure 5C**) and scanning electron microscope (SEM) images (**Figure 5D**). As tumor cells in BSA-coated microwells exhibited round morphology, NK cells mostly contacted them from the side and made horizontal interactions, whereas tumor cells in FN-coated microwells exhibited flat morphology, thus NK cells frequently stayed upside of the tumor cells and interacted vertically. Lastly, single cell level NK cell cytoxicity was evaluated by measuring the time for killing (**Figure 5E**), time from the initial NK-tumor contact to the onset of tumor cell death identified by PI incorporation in the tumor cells. For all tumor cells, NK cells BSA-coated microwells exhibited significantly faster cytotoxicity against tumor cells than NK cells in FN-coated microwells.

**Figure 6:**
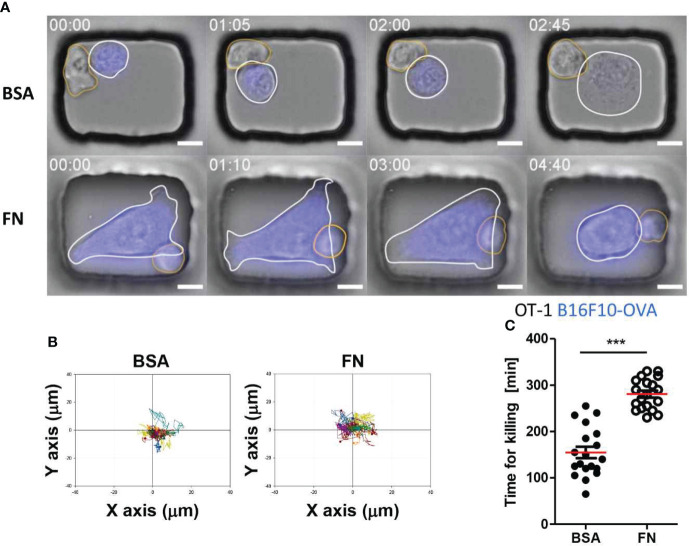
Dynamic behaviors and cytotoxicity of T cells in microwells coated with BSA or FN. **(A)** Representative time-lapse images of OT-1-B16F10-OVA interactions in microwells coated with BSA or FN. Scale bars: 10 μm. **(B)** Representative trajectories of T cell centroid. For each case, 15 T cells were traced over 5 h and plotted with the initial T-tumor contact points as the origin. **(C)** Effects of microwell surface coating on time for killing. Mann-Whitney test was used, *** < 0.001.

Identical experiments were performed for T cells using OT-1 T cells, which recognize ovalbumin-expressing tumor cells, and B16F10-OVA tumor cells, a melanoma cell line expressing ovalbumin. T cells in BSA-coated microwells behaved similarly to NK cells in that they maintained stable contact with round tumor cells (**Figure 6A** and **Movie S3** in SI). In contrast, T cells in FN-coated microwells formed stable contact on flat tumor cells with minimal translocation (**Figure 6A** and **Movie S4** in SI), entirely distinct from NK cells in FN-coated microwells, which continuously crawled on flat tumor cells. Individual T cell trajectories of demonstrated that T cells in both types of microwells did not migrate considerably from their initial interaction places with tumor cells (**Figure 6B**). Despite exhibiting identical dynamic characteristics, T cells in BSA-coated microwells killed tumor cells significantly faster than T cells in FN-coated microwells (**Figure 6C**).

Taken together, round tumor cells in BSA-coated microwells were more susceptible for killing by both T cells and NK cells than flat tumor cells in FN-coated microwells. These results suggest that the morphology of tumor cells determined by their adhesion microenvironments is a crucial factor in regulating immune cell-mediated cytotoxicity.

## Discussion

In this study, we devised microwell arrays coated with either cell repellent (BSA) or cell adhesion (FN) molecules to control tumor cell adhesion status, and to investigate whether tumor cell adhesion status influenced immune cell cytotoxicity. Solid tumor cells hardly adhere on the surfaces of BSA-coated microwells and exhibited a round morphology, whereas they firmly adhered to the bottom surfaces of FN-coated microwells with a flat morphology (**Figure 3**). By performing live cell imaging of one-to-one immune-tumor cell pairs, the detailed interaction dynamics of immune-tumor cell was observed, and the duration of interactions required for an immune cell to kill a tumor cell was measured at the single cell level (**Figures 5**, **6**).

Both NK cells and T cells demonstrated greater cytotoxicity against round tumor cells in BSA-coated microwells than against flat tumor cells in FN-coated microwells (**Figures 5E**, **6C**), indicating that adhering and spreading on the substrate itself may render solid tumor cells more resistant for killing by immune cells. This is the first demonstration that adhesion microenvironments of tumor cells influence immune cell cytotoxicity. Microwell arrays coated with BSA or FN made it possible to vary adhesion status of tumor cells while keeping most other experimental parameters, such as tumor cell types and cell density, constant and to directly observe immune cell mediated tumor killing in a single cell level, making them suitable for addressing this issue.

Detailed mechanisms underlying immune cells’ enhanced cytotoxicity against round tumor cells would be an interesting subject for future research. As identical solid tumor cells with different adhesion status were directly compared in this study, we speculate biophysical properties of tumor cells (31, 32), such as mechanical properties and curvatures, somehow influenced immune cell-mediated killing. Alternatively, the adhesion status of tumor cells may alter the expression of molecules to either promote or inhibit the activation of immune cells. We examined surface expression of an intercellular adhesion molecule 1 (ICAM-1), a key adhesion molecule for both T cell and NK cell synapses (33), and found that their expression levels and surface densities were not significantly influenced by the adhesion status of tumor cells (**Figure S1** in SI). To identify tumor cell adhesion status-dependent molecules that may either promote or inhibit immune cell-mediated killing, an extensive screening process would be necessary.

Immune cells kill tumor cells by either directly releasing lytic granules to tumor cells or engaging death receptors expressed on the surface of tumor cells to induce apoptosis (34). Typically, lytic granule-mediated killing is much faster than death receptor-mediated killing (35, 36), and NK cells first utilize lytic granules, and then switch to death receptors when lytic granules are depleted during serial killing (37). As we primarily assessed immune cell cytotoxicity in an early stage when immune cells encountered their first targets, lytic granule-mediated cytotoxicity was probably the predominant mode of killing in our study. Visualization of lytic granule dynamics revealed that NK cells interacting with round tumor cells in BSA-coated microwells converged and polarized lytic granules to the immune synapse much quickly than the NK cells interacting with flat tumor cells in FN-coated microwells (**Figure S2** in SI), implying that the adhesion status of tumor cells influenced the early stage of tumor cell recognition by immune cells (38). Importantly, an enhanced cytotoxicity was observed against the round tumor cells suspended in tubes compared to the flat tumor cells attached on substrates when NK cells were treated with concanamycin A (CMA) which blocks lytic granule-mediated cytotoxicity (39) (**Figure S2** in SI). This result suggests that immune cell-mediated cytotoxicity is influenced by the adhesion status of tumor cells regardless of the killing mechanisms of immune cells.

Both NK cells and T cells in BSA-coated microwells formed stable cell-cell conjugates with round solid tumor cells (**Figures 5A**, **6A**), similar to their interactions with blood cancer cells frequently observed during *in vitro* cytolytic synapse studies (40–42). In contrast, NK cells in FN-coated microwells predominantly climbed up on flat solid tumor cells and migrated continuously on them (**Figure 5A**), whereas T cells in FN-coated microwells maintained stable contact with flat solid tumor cells with low translocation (**Figure 6A**). Indeed, the Bousso group reported similar cellular dynamics in their intravital microscopy of mouse tumor tissues (43): NK cells exhibited dynamic motility during their tumor cell recognition and killing, whereas T cells contacting tumor cells were predominantly stationary. Given that solid tumor tissues are abundant in various types of extracellular adhesion molecules (44), these distinct behaviors are likely attributable to differences between NK cells and T cells in their exposure to extracellular adhesion molecules, in particular FN, during target cell recognition. Importantly, extracellular FN substantially altered NK-tumor cell interactions while having a negligible effect on T-tumor cell interactions. This discrepancy is likely attributable to differences in decision-making systems between NK cells and T cells. T cell receptor (TCR)-mediated signals play a predominate role in determining T cell behaviors (45); consequently, a strong TCR-mediated ‘stop’ signal is likely to override FN-induced ‘go’ signal. In contrast, NK cells express panels of receptors and weigh their signals relatively evenly when making decisions (46). Therefore, it is possible that extracellular FN signals destabilized NK-tumor cell synapses, similar to other NK inhibitory receptors (47, 48), to reduce NK cell cytotoxicity. Alternatively, extracellular FN can promote NK cells to surveil large area rather than focusing on a single target cell, and eventually promote serial killing of target cells (21).

The morphology of solid tumor cells in BSA-coated microwells is nearly identical to that of circulating tumor cells (CTCs) formed during metastasis, as both cell types share adhesion environments that prevent their firm attachment on substrates. Based on our observation that both NK cells and T cells demonstrated enhanced cytotoxicity against round solid tumor cells lacking substrate adhesion, they may serve as gatekeepers preventing tumor metastasis by efficiently killing CTCs in blood vessels before their colonization to other organs.

## Materials and methods

### Cell culture

A549 and PC-3 cells were cultured in RPMI 1640 media supplemented with 10% FBS and 1% penicillin/streptomycin. HeLa and B16F10-OVA cells were cultured in DMEM media supplemented with 10% FBS, 1% penicillin/streptomycin. NK-92 cells were cultured in MEM α supplemented with 12.5% FBS, 12.5% horse serum, 1% penicillin/streptomycin and 20 ng/mL of human IL-2.

OT-1 T cell were isolated and expanded from OT-1 T cell receptor transgenic mice. All experiments were performed according to a protocol approved by the institutional animal care and use committees of Seoul National University. On day 0, cells from spleen and lymph nodes of OT-1 mice were stimulated with 1 μg/ml OVA 257-264 peptide (SIINFEKL, Peptron,Inc., Korea) and cultured in RPMI 1640 media supplemented with 10% FBS and 1% penicillin/streptomycin, and 50 μM of beta-mercaptoethanol (sigma). On day 2, 5 ng/ml murine IL-2 (Peprotech) was added to the cells. Cells on day 4 were used for experiments.

### Flow cytometry-based cytotoxicity assay

A549, PC-3 and HeLa cells were harvested and suspended in serum-free medium, labeled with CFSE at 37 °C or 10 min, and the CFSE-labeled tumor cells were washed with serum-containing medium. To assess NK cell cytotoxicity against suspended tumor cells, the CFSE-labeled tumor cells were placed in 5mL Falcon round-bottom polystyrene tube (5 × 10^4^ cells/tube) and mixed with the same number of NK-92 cells at 37 °C or 4 h. To assess NK cell cytotoxicity against surface adhering tumor cells, the CFSE-labeled tumor cells were plated in a flat-bottom 96 well plates (5 × 10^4^ cells/well), incubated for 3 h to allow tumor cells adhere, the same number of NK-92 cells were added to the plates and incubated at 37 °C or 4 h, and the cell mixtures were harvested. PI was added to the cell mixture to label dead cells, and flow cytometry was performed using LSR Fortessa (BD Bioscience). Data was analyzed using FlowJo (FlowJo, LLC). Cytotoxicity were measured by the percentage of PI^+^ cells among CFSE^+^ cells.

### Tumor cell loading in microwell

Microwell arrays on glass coverslips were fabricated as previously described (49–51) by replicating silicon masters fabricated by standard photolithography twice. An array of rectangular microwells with a dimension of 50 μm × 45 μm was fabricated on a silicon wafer using a negative photoresist SU-8 50. The silicon master was treated with trichloro(1H,1H,2H,2H-perfluorooctyl) silane (Sigma Aldrich), and replicated using poly (dimethyl siloxane) (PDMS) by pouring PDMS precursor mixture (Sylgard 184, base: curing agent = 10: 1) and curing at 70°C for 4 h. The patterned PDMS mold was placed on a glass coverslip functionalized with acrylate group, perfused with a precursor solution (20% (v/v) poly (ethylene glycol) dimethacrylate (PEGDMA, Mn 750 Da) and 1% (w/v) 1-hydroxycyclohexyl phenyl ketone in 70/30 ethanol/water mixture) and cured with a UV for 1 min. The microwell array was extensively washed with deionized water for 3 ~ 5 days.

For surface coating, microwell arrays were treated with air-pasma (100 W, Femto Science, Korea) for 1 min, and incubated in protein solution containing either FN (2.27 μM, Sigma) or BSA (2.27 μM, Sigma) at 37 °C for 1 h. Tumor cell suspensions with various cell densities labeled with CellTrace Far Red were applied on the protein-coated microwell arrays, and briefly centrifuged to facilitate tumor cell loading in the microwells (1500rpm, 2 min). The FN-coated microwell arrays were incubated in in humidified incubator maintaining 37°C with 5% CO_2_ for 3 h to allow tumor cells to spread on the surface.

### Live cell imaging of immune cell-tumor cell interactions using fluorescence microscope

A modified Olympus IX 83 epi-fluorescence microscope with a 40X (UPlanFLN, NA=1.30) objective lens and a ANDOR Zyla 4.2 sCOMS CCD camera was used for imaging. A U-LH75XEAPO Xenon lamp (75W, Olympus) and eGFP (EX BP470/40, BS 495, EM/BP 525/50), Yellow (EX/BP 530/30, BS 550, EM/BP 575/40), Cy5 (EX/BP 620/60, BS 660, EM/BP 770/75) filter sets were used for fluorescence imaging. The microscope was automatically controlled by Micro-manager, and acquired images were analyzed and processed with Image J.

The microwell arrays loaded with the fluorophore-labeled tumor cells were mounted on magnetic chamber (Chamlide CF, Live Cell Instrument, Korea). Immune cell suspensions (either NK-92 or OT-1) containing 0.5 μM SYTOX Orange (Invitrogen), which labelled dead cells, were added. Subsequently, time-lapse imaging was initiated.

### Confocal microscopy

Tumor cells were labeled with 1 μM CFSE and NK-92 cells were labeled with CellTrace Far-red. The microwell arrays loaded with NK-92 cells and HeLa cells were maintained at 37 °C with 5% CO_2_ in humidified incubator for 2 hours. Then, DIC and fluorescence images of microwell arrays loaded with NK-92 and HeLa cells were acquired using a confocal laser scanning microscope (FV1200, Olympus) with a 20X (UPLSAPO 20X; NA =0.75) objective lens through optical z-stack (depth: 60 μm, interval 2μm). The acquired images were processed and analysed using ImageJ fiji and FV10-asw Viewer (Olympus).

### Scanning electron microscopy

Cells were fixed in Karnovesky’s fixative (0.1M cacodylate, 10% paraformaldehyde, and 8% glutaraldehyde in distilled water (DW)) at 4°C for 2 h. After washing the fixed cells with 0.05M sodium cacodylate buffer three times, they were further fixed in the second fixative (1% osmium tetroxide in 0.1M sodium cacodylate buffer) at 4°C for 1 h. Then, the fixed cells were washed three times with DW, and dehydrated in a series of ethanol solutions (from 30% to 100%) and finally in hexamethyldisilazane. Finally, cells were air-dried, coated with Pt, and observed using SUPRA 55VP (Carl Zeiss).

## Data availability statement

The original contributions presented in the study are included in the article/**Supplementary Material**. Further inquiries can be directed to the corresponding author.

## Ethics statement

The animal study was reviewed and approved by Institutional animal care and use committees of Seoul National University.

## Author contributions

S-EK and JD designed the study and developed the methodology. S-EK performed and analysed all the experiments. SY partially participated in the experiments. S-EK and SY prepared figures. S-EK and JD wrote the manuscript. All authors contributed to the article and approved the submitted version.

## Funding

This research was supported by the Ministry of Trade, Industry and Energy (MOTIE) and Korea Institute for Advancement of Technology (KIAT) through the International Cooperative R&D program (p0011266), the National Research Council of Science & Technology (NST) grant by the Korea government (CAP-18-02-KRIBB), and the National Research Foundation of Korea (NRF) grant (No. NRF-2020R1A2B5B03001747), Creative-Pioneering Researchers Program through Seoul National University

## Conflict of interest

The authors declare that the research was conducted in the absence of any commercial or financial relationships that could be construed as a potential conflict of interest.

## Publisher’s note

All claims expressed in this article are solely those of the authors and do not necessarily represent those of their affiliated organizations, or those of the publisher, the editors and the reviewers. Any product that may be evaluated in this article, or claim that may be made by its manufacturer, is not guaranteed or endorsed by the publisher.

## Supplementary material

Supplementary Video S1 A representative movie showing an NK-92 cell (yellow boundary) interacting with a HeLa cell (blue labeled with white boundary) in a BSA-coated microwell. Scale bars: 10 μm. Time stamp: hh:mm.

Supplementary Video S2 A representative movie showing an NK-92 cell (yellow boundary) interacting with a HeLa cell (blue labeled with white boundary) in a FN-coated microwell. Scale bars: 10 μm. Time stamp: hh:mm.

Supplementary Video S3 A representative movie showing an OT-1 cell (yellow boundary) interacting with a B16F10-OVA cell (blue labeled with white boundary) in a BSA-coated microwell. Scale bars: 10 μm. Time stamp: hh:mm.

Supplementary Video S4 A representative movie showing an OT-1 cell (yellow boundary) interacting with a B16F10-OVA cell (blue labeled with white boundary) in a FN-coated microwell. Scale bars: 10 μm. Time stamp: hh:mm.
