# Corrigendum: Effects of extracellular adhesion molecules on immune cell mediated solid tumor cell killing

**DOI:** 10.3389/fimmu.2022.1132806

**Published:** 2023-01-09

**Authors:** Seong-Eun Kim, Suji Yun, Junsang Doh

**Affiliations:** ^1^ Department of Mechanical Engineering, Pohang University of Science and Technology (POSTECH), Pohang, Gyeongbuk, Republic of Korea; ^2^ Interdisciplinary Program for Bioengineering, Seoul National University, Seoul, Republic of Korea; ^3^ Department of Materials Science and Engineering, Research Institute of Advanced Materials, Institute of Engineering Research, Bio-MAX Institute, Soft Foundry Institute, Seoul National University, Seoul, Republic of Korea

**Keywords:** cytotoxicity assay, single cell array, microwell assay, live cell imaging, extracellular adhesion molecules, NK cell, T cell, cancer immunotherapy

## Incorrect funding

In the published article, there was an error in the Funding statement. We missed grant number of “the Ministry of Trade, Industry and Energy (MOTIE) and Korea Institute for Advancement of Technology (KIAT) through the International Cooperative R&D program”. The correct Funding statement appears below.

