# Supplementary material for: Effects of extracellular adhesion molecules on immune cell mediated solid tumor cell killing
Source: Front Immunol. 2022 Oct 27;13:1004171. doi: 10.3389/fimmu.2022.1004171 (PMC9647090; doi:10.3389/fimmu.2022.1004171)
Supplement: Supplementary file 5 [file DataSheet_1.pdf]

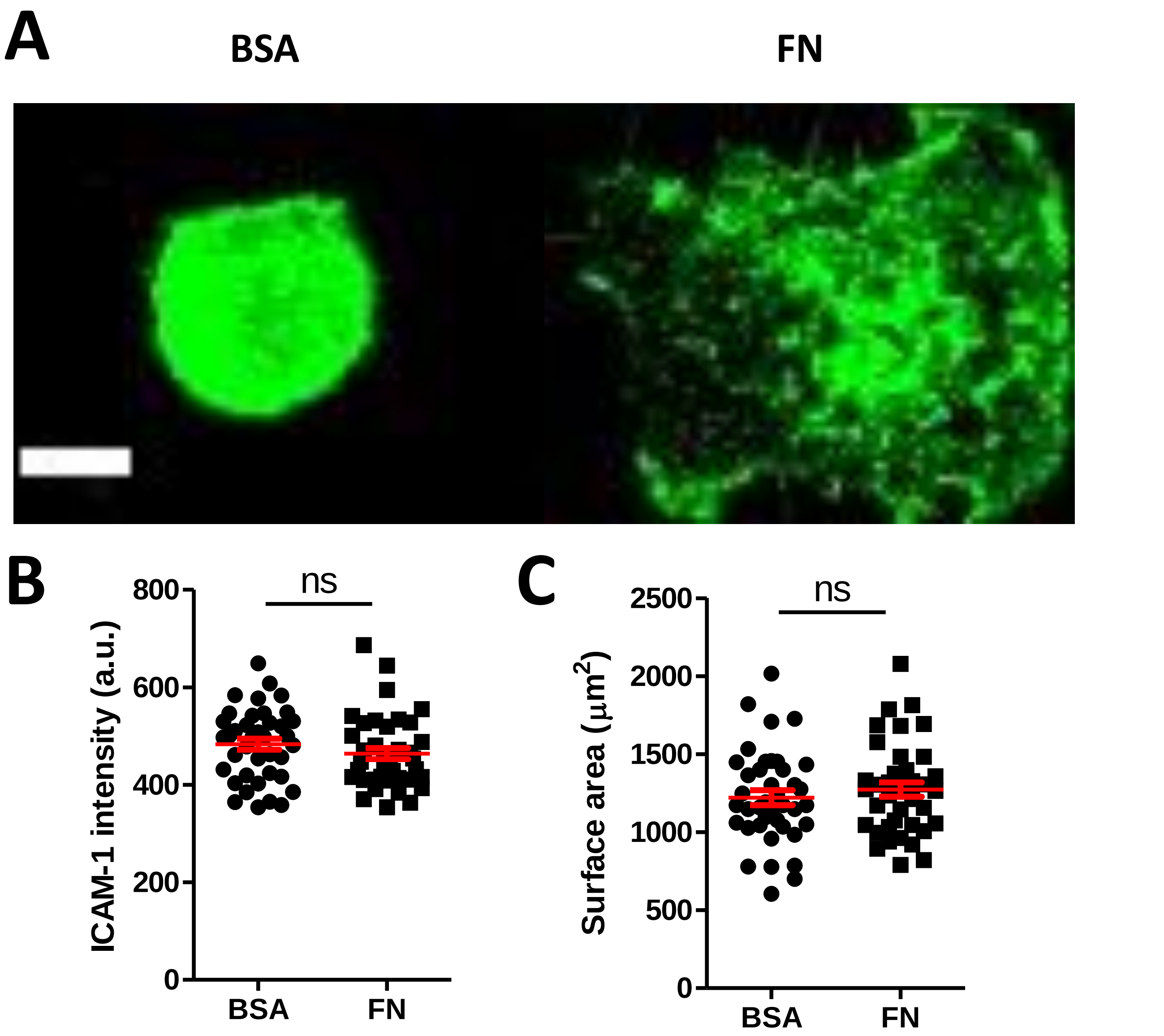

Figure S1. Intercellular adhesion molecule 1 (ICAM-1) expression of tumor cells in BSA- and FN-coated microwells. A. Confocal images of ICAM-1-stained tumor cells. Scale bar: 10 mm. **B-C.** Quantification of ICAM-1 fluorescence intensities **(B)**, and surface areas **(C)** of tumor cells in BSA- and FN-coated microwells. HeLa cells in either BSA- or FN-coated microwells were fixed with 4% paraformaldehyde, and stained with Alexa Fluor 488 anti-human ICAM-1 (Biolegend). ICAM-1 intensities in 'B' were calculated by integrating fluorescence intensities of all optical z-section images acquired by confocal microscopy. Surface areas of cells in 'C' were calculated using the measured diameter for cells in BSA-coated microwells, or directly measured for cells in FN-coated microwells. Mann-Whitney test was used, ns: not significant.

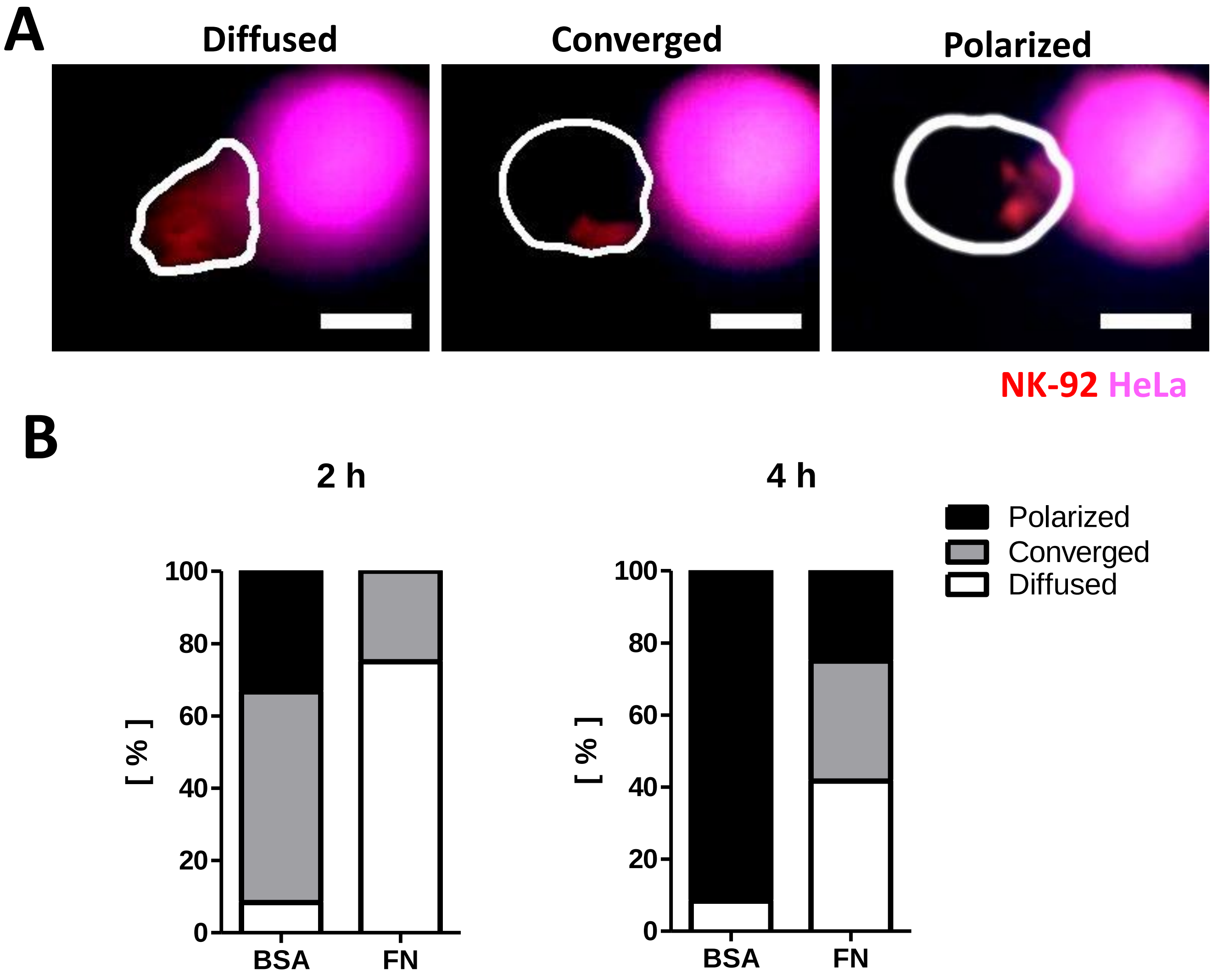

Figure S2. Lytic granule dynamics in NK cells interacting with tumor cells in BSA- and FN-coated microwells. A. Representative fluorescence images showing different stages of lytic granule distribution in NK cells interacting with tumor cells. Scale bar: 10 mm. **B.** Percentage of NK cells exhibiting different lytic granule distributions at 2 and 4 h after contacting tumor cells. NK-92 cells labeled with 1 mM LysoTraker Red (Invitrogen) and HeLa cells labeled with 1 mM CellTrace Far-red (Invitrogen) were used.

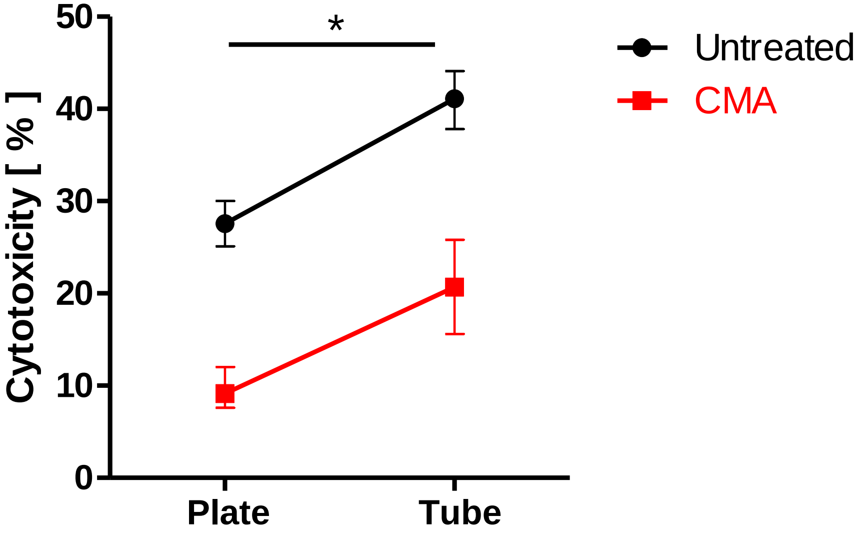

Figure S3. Effects of concanamycin A (CMA) treatment on NK cell-mediated cytotoxicity against tumor cells adhered on plates and suspended in tubes. NK-92 cells either pre-treated with 20 nM CMA for 2 h (CMA) or not (untreated) were added to CFSE-labeled HeLa cells either attached on flat-bottom well plates or suspended in round-bottom tubes, and incubated for 4 h in the presence of propidium iodide (PI). Cytotoxicity was assessed by harvesting cells from the plates or tubes and measuring percentage of PI-stained cells among CFSE-labeled cells by flow cytometry. Mann-Whitney test was used, *<0.05.
